# Caffeoylquinic Acids in *Centella asiatica* Reverse Cognitive Deficits in Male 5XFAD Alzheimer’s Disease Model Mice

**DOI:** 10.3390/nu12113488

**Published:** 2020-11-13

**Authors:** Donald G. Matthews, Maya Caruso, Armando Alcazar Magana, Kirsten M. Wright, Claudia S. Maier, Jan F. Stevens, Nora E. Gray, Joseph F. Quinn, Amala Soumyanath

**Affiliations:** 1 Department of Neurology, School of Medicine, Oregon Health & Science University, Portland, OR 97239, USA; matthedo@ohsu.edu (D.G.M.); carusma@ohsu.edu (M.C.); wrigkir@ohsu.edu (K.M.W.); grayn@ohsu.edu (N.E.G.); quinnj@ohsu.edu (J.F.Q.); 2 Department of Chemistry, Oregon State University, Corvallis, OR 97331, USA; alcazara@oregonstate.edu (A.A.M.); claudia.maier@oregonstate.edu (C.S.M.); 3 Linus Pauling Institute, Oregon State University, Corvallis, OR 97331, USA; fred.stevens@oregonstate.edu; 4 Department of Pharmaceutical Sciences, College of Pharmacy, Oregon State University, Corvallis, OR 97331, USA; 5 Parkinson’s Disease Research Education and Clinical Care Center, Veterans’ Administration Portland Health Care System, Portland, OR 97239, USA

**Keywords:** *Centella asiatica*, family Apiaceae, 5XFAD, conditioned fear response, triterpenes, caffeoylquinic acids

## Abstract

*Centella asiatica* (CA) is an edible plant and a popular botanical dietary supplement. It is reputed, in Ayurveda, to mitigate age-related cognitive decline. There is a considerable body of preclinical literature supporting CA’s ability to improve learning and memory. This study evaluated the contribution of CA’s triterpenes (TT), widely considered its active compounds, and caffeoylquinic acids (CQA) to the cognitive effects of CA water extract (CAW) in 5XFAD mice, a model of Alzheimer’s disease. 5XFAD mice were fed a control diet alone, or one containing 1% CAW or compound groups (TT, CQA, or TT + CQA) equivalent to their content in 1% CAW. Wild-type (WT) littermates received the control diet. Conditioned fear response (CFR) was evaluated after 4.5 weeks. Female 5XFAD controls showed no deficit in CFR compared to WT females, nor any effects from treatment. In males, CFR of 5XFAD controls was attenuated compared to WT littermates (*p* = 0.005). 5XFAD males receiving CQA or TT + CQA had significantly improved CFR (*p* < 0.05) compared to 5XFAD male controls. CFR did not differ between 5XFAD males receiving treatment diets and WT males. These data confirm a role for CQA in CAW’s cognitive effects.

## 1. Introduction

*Centella asiatica* (L.) Urban (CA), family Apiaceae, also known as Indian Pennywort, Mandukaparni, or Brahmi is an edible herb and popular botanical dietary supplement. It is an important Ayurvedic herb used for memory improvement [1]. The cognitive and neurological benefits of CA have been widely studied in preclinical and clinical investigations [2]. For example, we demonstrated that a CA water extract (CAW) dose-dependently reversed memory deficits in novel object recognition and conditioned fear response (CFR) in both male and female 5XFAD transgenic mice, an amyloid-β-driven model of Alzheimer’s disease (AD) [3]. The identification of active compounds of CA is important, both to understand the mode of action of this botanical, and for possible standardization of herbal preparations of CA for memory enhancement. The characteristic triterpenes (TT) of CA (asiatic acid, madecassic acid, and their glycosidic forms) are generally considered to be the active compounds in CA. However, studies have shown that both TT and caffeoylquinic acids (CQA) found in CA have neurotropic and neuroprotective effects [2,4,5]. In this study, we treated male and female 5XFAD mice with CAW, or CAW-equivalent amounts of TT and CQA compounds, to explore the role of these two groups of compounds in the cognitive effects of CAW. Cognitive function was assessed using CFR, as in our earlier study on CAW [3].

## 2. Materials and Methods 

### 2.1. Preparation of CA Dried Water Extract (CAW)

CAW was prepared as previously described [3]. Briefly, dry *Centella asiatica* (CA) herb, obtained from Oregon’s Wild Harvest (Redmond, OR, USA; Lot # 170300206; 160 g) was boiled in distilled water (2 L) under reflux (2 h), filtered through 185 mm (grade 1) Whatman filter paper to remove plant material, and the extract lyophilized to yield ~24% dry CAW, which was stored at −20 °C until use. A voucher sample of the original plant material has been deposited at Oregon State University Herbarium (OSC-V-258629), while voucher samples of plant material and CAW (designated “CAW-iota”) are held in our laboratory. TT and CQA compounds in CAW-iota were quantified using liquid chromatography-high resolution tandem mass spectrometry (LC-HRMS/MS (Table 1).

### 2.2. CAW Individual Compounds

Compounds were purchased from TransMIT (Gießen, Germany; all compounds were analyzed by the manufacturer using HPLC with purity > 98%). TT compounds: asiatic acid (CAS 464-92-6), asiaticoside (CAS 16830-15-2), madecassic acid (CAS 18449-41-7), and madecassoside (CAS 34540-22-2); CQA compounds: 1,3-dicaffeoylquinic acid (CAS 19870-46-3), 1,5-dicaffeoylquinic acid (CAS 30964-13-7), 3-O-caffeoylquinic acid (chlorogenic acid) (CAS 327-97-9), 3,5-dicaffeoylquinic acid (isochlorogenic acid A) (CAS 2450-53-5), 3,4-dicaffeoylquinic acid (isochlorogenic acid B) (CAS 14534-61-3), and 4,5-dicaffeoylquinic acid (isochlorogenic acid C) (CAS 32451-88-0), 5-O-caffeoylquinic acid (neochlorogenic acid) (CAS 906-33-2). Compound identity was confirmed using LC-HRMS/MS and comparison against standards.

### 2.3. LC-HRMS/MS Analysis

Samples (10 mg) of CAW-iota, the standard rodent diet of our institution (PicoLab Laboratory Rodent Diet 5L0D, LabDiet, St. Louis, MO, USA), or Modified AIN-93M Purified Rodent diet (Dyets Inc, Bethlehem, PA, USA), were suspended in aqueous methanol (70% *v*/*v* with 0.1% of formic acid; 10 mL), sonicated (30 min, 25 °C), and centrifuged (14,000× *g*, 10 min). Supernatants were analyzed using a validated LC-HRMS/MS method [6]. Individual TT and CQA were quantified using calibration curves constructed with reference compounds. 

### 2.4. Experimental Mouse Diets

Due to limited availability of purified compounds, CAW and compounds were administered in the diet rather than drinking water, as used in our previous study [3]. Test materials were incorporated into AIN-93M diet by Dyets Inc. (Bethlehem, PA, USA). CAW was incorporated at 1% w/w to approximate the maximum 1000 mg per kg of body weight per day (mg/kg/d) dose used in our previous dose-response study [3]. TT and CQA were incorporated into AIN-93M to match their concentration (*w*/*w*) in the 1% CAW diet (Table 1). Diets included a TT mixture (asiatic acid, asiaticoside, madecassic acid, and madecassoside), a CQA mixture (1,3-dicaffeoylquinic acid, 1,5-dicaffeoylquinic acid, chlorogenic acid, isochlorogenic acid A, isochlorogenic acid B, isochlorogenic acid C, neochlorogenic acid), and a combined diet of all these compounds (TT + CQA). To ensure homogenous distribution, the relatively small amounts of CAW or test compounds were first premixed with the larger sucrose or vitamin components of the AIN-93M diet, and then combined with the other required ingredients until uniform. Cold water (10%) was added for pelleting, and the diet run through a California Pellet Mill CL-3 lab pellet mill. The pellets were placed on trays and air dried using dry, warm air at 27 °C for 24 h. Diets were sterilized by gamma irradiation (5.0–20.0 kGy) at Sterigenics (Oak Brook, IL, USA).

### 2.5. Animals

All experiments were conducted according to NIH Guidelines for the Care and Use of Laboratory Animals, and approved by the Institutional Animal Care and Use Committee of the Portland VA Medical Center (IACUC #: 3260-17). C57BL/6:SJL F1 female mice (Jackson Laboratory, Sacramento, CA, USA) were bred with 5XFAD male mice. 5XFAD and wild-type (WT) littermate progeny were genotyped via PCR of transgenic hAPP from DNA tail samples. Animals were housed (1–4 mice per cage) on an alternating 12-h light–dark schedule, in a climate-controlled environment. Food and water were given ad libitum. Male and female 5XFAD mice (7.7–8.5 ± 0.1 months of age) were taken off the standard diet and fed AIN-93M (vehicle diet), or AIN-93M containing 1% CAW or CA compound mixtures (TT, CQA, or TT + CQA) for a total of 4.57 ± 0.01 weeks (end of behavioral testing), while WT littermates received only the AIN-93M vehicle diet (total of 12 groups, *n* = 12–24). Animal weights were recorded (females: 28.9 ± 0.5 g; males: 29.3 ± 0.4 g) and diet consumption (females: 3.4 ± 0.1 g/d; males: 3.5 ± 0.1 g/d) estimated every 3–4 days from the remaining food, for the duration of the treatment.

### 2.6. Conditioned Fear Response (CFR)

CFR experiments were performed starting during the 5th week of treatment. After a 5-min habituation period where baseline freezing was measured (ANY-Maze Software, Stoelting Co., Wood Dale, IL, USA), three consecutive 1-min periods were observed, as one shock per period was randomly applied (Fusion v6.0 for SuperFlex Edition Software, Omnitech Electronics, Inc., Columbus, OH, USA). At 24 h after shock treatment, contextual fear freezing during a 5-min period was measured, and normalized by subtracting the freezing time observed during the habituation period for each mouse.

### 2.7. Statistical Analysis

All treatment comparisons were statistically evaluated using one-way ANOVA, and Tukey’s HSD as the post-hoc multiple comparisons test (threshold *p* < 0.05). Sex differences within treatments were evaluated using Student’s t-test (threshold *p* < 0.05). Statistical analyses were performed using R statistical analysis software (version R 3.6.2; R Foundation for Statistical Computing, Vienna, Austria).

## 3. Results

TT and CQA content in CAW was determined by LC-HRMS/MS (Table 1). Diets containing TT, CQA, or TT + CQA were prepared to be equivalent to their contents in the 1% CAW diet. AIN-93M was used as the vehicle and control diet in this study, as it did not contain detectable amounts of the TT or CQA of interest (Table 1), whereas CQA compounds were present in small amounts in the standard mouse diet used in our laboratory from weaning until the time of treatment (PicoLab Laboratory Rodent Diet, 5L0D; LabDiet, St. Louis, MO, USA). Every precaution was taken to minimize degradation of CAW compounds in the AIN-93M treatment diets during storage (stored at 4 °C in the dark prior to administration). 

Daily consumption of the diets was estimated from the average daily food intake and the average body weight of male and female mice (Table 2). Although the diets were designed to deliver TT and/or CQA equivalent to the CAW group, 5XFAD females consumed approximately 20% more of the 1% CAW diet than the other diets (*p* < 0.001), and a greater amount of the 1% CAW diet (*p* = 0.014), and lower amount of the CQA diet (*p* = 0.032) than 5XFAD males. Male mice consumed similar amounts of all treatment diets. The calculated dose of CAW was 1370 ± 40 mg/kg/d for females and 1220 ± 40 mg/kg/d for males, which fell within the range of our previous dose-response study [3]. 

**Figure 1:**
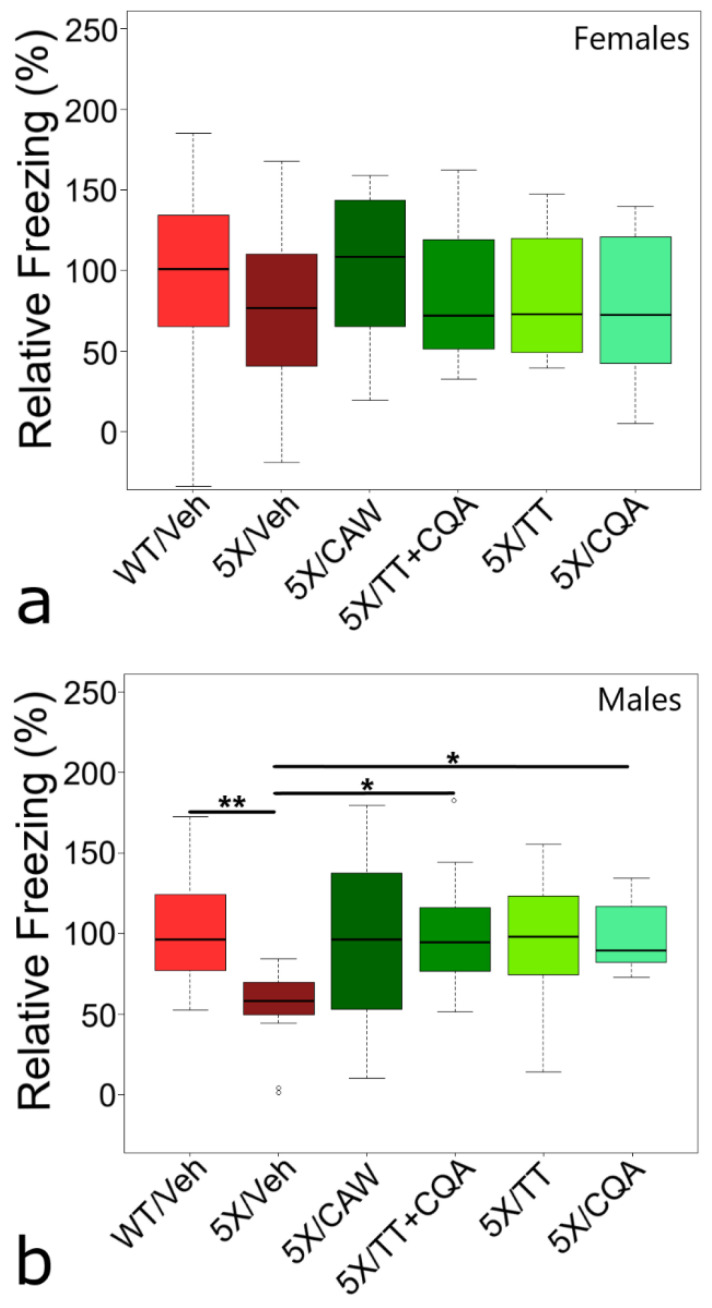
Conditioned fear memory evaluation of 5XFAD mice (5X) after treatment with *Centella asiatica* water extract (CAW) or CAW compounds. (**a**,**b**) Contextual fear response (CFR) analysis after 4 weeks of treatment being incorporated into the diet. (**a**) Neither genotype nor treatment had an impact on memory in 5XFAD females. Vehicle-treated (Veh) wild-type (WT) females froze for 141.6 ± 14.7 s and was set to 100%. (**b**) 5XFAD males demonstrated a memory deficit compared to WT males (decreased 46.8%, *p* = 0.005). Treatment of 5XFAD males with triterpenes (TT) + caffeoylquinic acids (CQA) (*p* = 0.024) or CQA (*p* = 0.039) improved memory, and treatment with CAW (*p* = 0.095) and TT (*p* = 0.082) had non-significant improvements. Vehicle-treated WT males froze for 131.2 ± 9.2 s and was set to 100%. Analysis of CFR data determined by ANOVA analysis, followed by Tukey post-hoc testing with * *p* < 0.05, ** *p* < 0.01. Group sizes for females (**a**) were WT control, 5XFAD control, CAW, TT + CQA, TT, CQA: *n* = 24, 21, 13, 12, 12, 15, and for males (**b**) were WT control, 5XFAD control, CAW, TT + CQA, TT, CQA.

Contextual memory was tested using a CFR paradigm. The small CFR deficit in 5XFAD females compared to WT littermates (24.3% reduction in mean freezing time), failed to reach statistical significance (*p* = 0.508) (Figure 1a). This could be a consequence of the stronger fear response in female than male mice [7]. The lack of any treatment effects on memory in females was unexpected, as CAW administered in drinking water previously improved CFR in both WT and 5XFAD females [3].

Male 5XFAD mice (Figure 1b) showed a significant (*p* = 0.005) deficit in CFR (down 46.8%) vs. male WT littermates. Compared to male 5XFAD mice on vehicle diet, significant improvements in CFR were seen in these mice after treatment with diets containing CQA alone (82% increase, *p* = 0.039) or in combination with TT (TT + CQA – 87% increase, *p* = 0.024). All diets containing CAW, CQA, and/or TT, improved CFR in male 5XFAD mice, to levels not significantly different from male WT littermates (92–99% of vehicle-treated WT males *p* = 0.987–1.000).

## 4. Discussion

CFR improvement seen following CQA treatment in these male 5XFAD mice was consistent with our earlier in vitro studies demonstrating a neuroprotective effect of CQA compounds against Aβ toxicity [4], as well as with our study showing beneficial effects of CQA compounds on dendritic arborization and spine density in primary neurons [5]. Our findings are likewise in line with other reports suggesting that CQA improves memory and cognition in rodent ageing models [2,8,9]. TT treatment has also been shown to improve cognitive function in rodent models, making the non-significant effect of TT treatment in this study somewhat surprising [10,11]. This difference may be due to the lower TT doses and treatment duration used in the present study.

Factors other than memory may have contributed to the CFR results, including changes in caloric intake, locomotor activity, or anxiety. We do not consider food restriction to have been a factor in the CFR results as each group averaged more than 3 g of diet per day per mouse, and body weights were monitored throughout the experiment (no persistent weight loss was observed).

Even though 5XFAD mice are reported to have some motor dysfunction at 9 months of age [12,13], we did not observe a strong increase in freezing (i.e., no decreased movement), during habituation in untreated 5XFAD males versus untreated WT males (*p* = 0.093). In fact, there was a decrease in untreated 5XFAD male freezing (i.e., more movement) during the 5 min of CFR evaluation (vs. untreated WT males, *p* = 0.042). We also did not observe a significant increase in 5XFAD movement during habituation with any treatment in males (*p* = 0.157). Therefore, alteration of locomotor activity by CAW or its components is unlikely to have played a significant role in the CFR results in male mice. On the other hand, untreated female 5XFAD mice demonstrated decreased movement during habituation (vs. untreated WT females, *p* = 0.003) which was alleviated by 1% CAW treatment only (vs. untreated 5XFAD females, *p* = 0.013). These changes in habituation movement may have influenced the normalized CFR freezing values in females (Figure 1a). As a result, further testing of both memory and locomotor efficiency should be addressed in future experiments involving CAW treatment of female 5XFAD mice.

CA extracts and their active compounds have known anxiolytic effects [14,15,16]. Reducing anxiety during the CFR test should either reduce fear acquisition [17,18] or have no effect [19]. However, in this study we observed an increase in fear response with all treatments in male 5XFAD mice. Therefore, we are confident that the prevailing influence on CFR freezing is indeed contextual fear memory and not on anxiety in male 5XFAD mice. As no differences in fear response were detected in females with genotype or treatment, further investigation is required to determine if anxiety plays a role in CFR testing in 5XFAD females treated with CAW or its active compounds.

We have shown that CAW treatment improves the performance of WT and/or AD model mice in multiple other behavioral tests including the Morris water maze [20,21], novel object recognition [3,22], object location memory [22,23], and odor discrimination learning reversal [22,23] paradigms, demonstrating an influence on hippocampal- and cortical-dependent memory as well as prefrontal cortex-mediated executive function. It will be important to evaluate the effects of the TT and CQA components of CAW in mice using each of these paradigms, in order to obtain a more comprehensive picture of their pro-cognitive effects, including potential differences in the cognitive functions modulated by each group of components.

The presence of a significant CFR deficit (compared to WT mice) only in male, and not female, 5XFAD mice was unanticipated, given our earlier study where a clear deficit was observed in female 5XFAD mice tested at about 8 months of age [23]. It is unclear if the different result here was due to the greater age of the mice (9 months), or to unknown, cohort-specific factors. Sex differences in the 5XFAD model at the biochemical level have been studied by our group [3] and others [24,25,26,27]. Previously, we have illustrated that a CAW-mediated improvement of WT and 5XFAD memory in both sexes may be due to mitigation of oxidative stress as illustrated by an increase in NRF-2-regulated gene expression in the hippocampus [3,23]. This reduction in oxidative stress after CAW treatment could be the reason for the reduction in SOD1 protein in the CAW-treated 5XFAD hippocampus [3]. Furthermore, in a dose-responsive manner to CAW treatment, hippocampal gene expression of synaptophysin and *Psd95* increases, indicating a possible synaptic health benefit of CAW, particularly in 5XFAD males, which saw elevated levels of both synaptic markers versus females [3]. In a related study [23], hippocampal mitochondrial function, and hippocampal and cortical expression of mitochondrial genes were increased by CAW treatment in 5XFAD female mice (males were not evaluated). Brain-derived neurotropic factor (BDNF) may mediate the cognitive effects of CA. Rats treated with complex extracts of CA enriched in TT showed improved cognition and elevated *Bdnf* mRNA in the prefrontal cortex [28], or BDNF protein in the hippocampus [29]. The CQA content of the CA extracts was not reported. Further studies are warranted to separately elucidate the role of BDNF in the cognitive effects of CA’s TT and CQA.

The important role of CQA compounds in facilitating the cognitive benefits of CAW is clear in this study, with the significant improvement of male 5XFAD response to conditioned fear in both the CQA and TT + CQA treatment groups. Hitherto, standardization of CA preparations has mostly focused on the TT components, due to the more extensive literature on their neuroprotective and neurotropic effects [2]. The present study demonstrates that CQA content must also be considered when standardizing CA preparations for use in phytotherapy or clinical trials.

The muted impact of CAW on behavior in both male and female 5XFAD mice in the present study was surprising given the cognitive effects we had previously observed, even at lower CAW doses [3]. This difference may be a consequence of administering CAW incorporated in the diet rather than drinking water. In future studies, it would be useful to compare the stability of CAW compounds in drinking water with stability during preparation, storage, and administration in a diet. The mode of administration may also influence availability, and absorption of the active compounds. These processes may vary with gut transit time of the preparation, or gut microbial transformation, which in turn may be influenced by gender differences. The observations regarding sex differences in response to CAW (and TT and CQA), and the effect of changing the mode of administration from drinking water to diet, warrant further exploration, as they may also be relevant to the clinical use of CA preparations.

## Figures and Tables

**Table 1:** LC-HRMS/MS quantification of phytochemicals in vehicle mouse diets and *Centella asiatica* water extract (CAW).

Compound	Compounds in Extract (mg/g ± SEM)	Compounds in Diet (µg/g ± SEM)		
	CAW Extract	Standard Diet	AIN-93M Diet	AIN-93M Diet + 1% CAW Extract Calculated
**Caffeoylquinic acids**				
1,3-dicaffeoylquinic acid	0.67 ± 0.01	<LOQ	<LOQ	6.7
1,5-dicaffeoylquinic acid	0.64 ± 0.01	<LOQ	<LOQ	6.4
Chlorogenic Acid	5.25 ± 0.02	9.3 ± 0.1	<LOQ	52.5
Isochlorogenic Acid A	2.29 ± 0.01	9.5 ± 2	<LOQ	22.9
Isochlorogenic Acid B	3.60 ± 0.02	5.5 ± 2	<LOQ	36.0
Isochlorogenic Acid C	2.64 ± 0.01	9.7 ± 2	<LOQ	26.4
Neochlorogenic Acid	1.49 ± 0.01	2.2 ± 0.1	<LOQ	14.9
Total Caffeoylquinic Acids	16.57 ± 0.04	36.2 ± 3	<LOQ	165.7
**Triterpenes**				
Asiatic acid	0.57 ± 0.01	<LOQ	<LOQ	5.7
Asiaticoside	23.87 ± 1.72	<LOQ	<LOQ	238.7
Madecassic Acid	0.94 ± 0.02	<LOQ	<LOQ	9.4
Madecassoside	18.64 ± 1.84	<LOQ	<LOQ	186.4
Total Triterpenes	44.01 ± 2.52	<LOQ	<LOQ	440.1

SEM derived from analysis in triplicate. <LOQ (Below Limits of Quantitation). Values for the “AIN-93M diet + 1% CAW extract” were calculated based on their content in CAW extract.

**Table 2:** Analysis of dosing in all treatment groups.

	Food Consumption Rate (g/kg body weight/d)		Sex Comparison
	Females	Males	
Treatment	Avg ± SE	Avg ± SE	*p*-Value
Vehicle ^†^	128 ± 2 ^bc^	130 ± 3	0.541
1% CAW	137 ± 4 ^c^	122 ± 4	0.014
CQA	111 ± 4 ^a^	123 ± 4	0.032
TT	112 ± 4 ^a^	118 ± 2	0.203
TT + CQA	120 ± 4 ^ab^	125 ± 6	0.504

Average (Avg) food consumption (and standard error (SE)) was calculated relative to body weight per day. No dosing differences between diets were observed in the male groups (*p* = 0.14). Females on 1% *Centella asiatica* water extract (CAW) diet consumed more diet compared to all other treatment groups (vs. triterpenes (TT)–*p* < 0.001, vs. caffeoylquinic acids (CQA)–*p* < 0.001, vs. TT + CQA–*p* = 0.022). Females on the vehicle diet consumed more than 5XFAD females on CQA (*p* = 0.001) or TT (*p* = 0.009), but not more than 1% CAW (*p* = 0.264) or TT + CQA (*p* = 0.398). Females consumed more 1% CAW diet than males (*p* = 0.014), and less CQA diet than males (*p* = 0.032). Letters (^abc^) represent statistical similarity in consumption within a sex, as determined by ANOVA analysis, followed by Tukey post-hoc testing. ^†^ Vehicle diet consumption contains data from combined wild-type (WT) and 5XFAD groups housed together.
